# Teaching landscape of robotic anatomic lung resection: an analysis of the da Vinci system dual console use in the US

**DOI:** 10.1007/s11701-025-02976-0

**Published:** 2025-12-05

**Authors:** Neelesh Bagrodia, Ammu V. Alvarez, Mahmoud Abdel-Rasoul, Rezzan Hekmat, Robert E. Merritt, Gretchen P. Jackson, Peter J. Kneuertz

**Affiliations:** 1 Thoracic Surgery Division, Department of Surgery, The Ohio State University, Columbus, OH USA; 2 Center for Biostatistics, Department of Biomedical Informatics, The Ohio State University Wexner Medical Center, Columbus, OH USA; 3 Digital, Intuitive Surgical, Sunnyvale, CA USA; 4 Department of Pediatric Surgery, Vanderbilt University Medical Center, Nashville, TN USA; 5 Division of Thoracic Surgery, The Ohio State University Wexner Medical Center, 410 W 10th Ave, Columbus, OH 43210 USA

**Keywords:** Robotic surgery, Surgical education, Minimally invasive thoracic surgery

## Abstract

We sought to characterize the utilization of the dual console (DC) teaching setup for daVinci robotic-assisted anatomic lung resections on a US national level. Intuitive da Vinci robotic system data for thoracic lobectomy and segmentectomy procedures between 2021 and 2022 were included. DC utilization was categorized as never, low, medium or high for surgeons performing 0, < 50%, 50–90% or > 90% of resections with DC activation, respectively. Surgeons and procedure factors associated with DC use was analyzed using binary logistic regression. During the study period, 38,552 lobectomy and 4,955 segmentectomy procedures were performed by 868 unique surgeons. A total of 555 surgeons (63.9%) performed anatomic lung resections using the DC, whereas 313 surgeons (36.1%) never used the DC. Amongst DC users, 176 (31.7%) surgeons were classified as low, 216 (38.9%) as medium and 163 (29.3%) as high utilizers. DC use varied significantly between surgeons based on experience (*p* = 0.018), with the greatest proportion of high utilizers being surgeons with less than 5 years of experience on the robot. Overall, the DC was used for 42.9% of anatomic lung resection. DC use was higher for segmentectomy than for lobectomy procedures (54.9% vs. 41.4%; *p* < 0.001). The median console time was 18 min longer with the DC setup (123.9 vs. 105.1 min, *p* < 0.001). On multivariable analysis, stapler use and Academic or VA/DOD hospital setting were associated with the highest rates of dual console activation. The dual console teaching setup is being used by almost two thirds of surgeons in the US. However, the significant variation of dual console activation indicates a wavering level of participation in teaching amongst surgeons. Further research is needed on robotic trainee involvement and pathways to autonomy for the next generation of thoracic surgeons.

## Introduction

Robotic surgery has revolutionized the way many surgeons operate in various disciplines. In the field of thoracic surgery, the da Vinci robotic system (Intuitive Surgical Inc., Sunnyvale, CA) has been successfully applied to pulmonary resections, mediastinal procedures, and esophageal surgery, allowing for equivalent oncologic outcomes while reducing complications, decreasing hospital stay and shortening recovery times as compared to traditional open surgery [1]. The learning curve is steep, especially for surgeons with existing video-assisted thoracic surgery (VATS) skills, but conventional hands-on training methods do not apply. Unlike open surgery, where an attending surgeon can directly guide a trainee’s hands while operating together, robotic surgery is typically performed by one surgeon who maintains full control of the instruments and the camera [1]. 

The dual console setup is used in residency and fellowship training programs to facilitate training on the da Vinci system; it involves two consoles through which control of an operation can be shared. An experienced surgeon can supervise trainees in real-time and instantly take control if necessary, ensuring patient safety. Dual consoles are also used for proctoring surgeons who are newly adopting robotic techniques [1]. As such, the dual console setup allows for real time guidance and is considered an essential component for any effective robotic training program [2]. However, little is known about how often the dual console gets used and how much it contributes to the teaching landscape.

The objective of this study was to characterize the use of the dual console system for robotic thoracic surgery on a US national level to better understand where it is used and how often surgeons utilize the platform.

## Methods

We utilized the Intuitive da Vinci Surgical System data of anatomic lung resections performed between 2021 and 2022 in the US. We chose anatomic lung resections to investigate as it is the most common type of operation performed in robotic thoracic surgery [1]. The study leveraged a protocol for analyses of da Vinci Surgical System data, which was deemed exempt by the WCG Institutional Review Board (Protocol# ISI 20221) on November 21, 2022.

### Data source and definitions

We analyzed a limited dataset derived from the Intuitive da Vinci Surgical System, from which surgeon, patient, and institutional identifiers were removed. To protect privacy and to minimize the risk of identifying surgeons or sites, we excluded data from surgeons who had conducted fewer than 10 cases. Surgeon data were attributed to the primary surgeon. Surgeon-specific information included primary surgical specialty (i.e., thoracic, cardiac, or general), year of first da Vinci robotic system use, advanced training completion, total and thoracic case volumes prior to 2022, practice setting, and geographic region. For each case, data collected included the da Vinci system model used, console time, use of the robotic stapler, and the number of stapler firings. A teaching case was defined as any case in which as dual console (DC) was active, irresective of the login status of the second console surgeon.

### Statistical analysis

Dual console system usage was categorized as follows: **No usage** were defined as surgeons who use the DC in 0% of cases; **low usage** as surgeons who utilize the DC in < 50% of total cases; **medium usage** as surgeons who utilize the DC for 50–90% of cases; **and high usage** for surgeons who use DC system in > 90%. Surgeon and procedure factors associated with dual console use were analyzed using binary logistic regression models. A multivariable model was created by backward stepwise selection to evaluate for factors with the strongest independent association with dual console use. Data analysis was performed by an experienced statistician (MAR) using SAS version 9.4 (SAS Institute, Cary NC) software.

## Results

### General characteristics of cases by dual console use

A total of 43,507 robotic anatomic lung resection procedures were analyzed, of which 18,664 (42.9%) incorporated DC use and 24,843 (57.1%) were performed using a single console (Table 1). Case length was longer for cases with DC activation, 136 ± 72 versus 116 ± 67 min (*p* < 0.001). The activation of the DC was higher for segmentectomy than for lobectomy procedures (54.9% vs. 41.4%; *p* < 0.001). The DC was used in most anatomic lung resections performed at Academic/Teaching hospitals (76.0%) or Department of Defense/Veterans Affairs hospitals (87.3%) and less than quarter of cases performed in the community hospital setting (24.6%; *p* < 0.0001).

**Table 1:** Procedure details of anatomic lung resection performed using the davinci robot in the US with dual versus single console

Procedure Details	Total (*n* = 43,507)	Dual Console Use (*n* = 18,664)	Single Console Use (*n* = 24,843)	*p*-value
**Procedure type**				< 0.001
Lobectomy	38,495 (88.5%)	15,923 (85.3%)	22,572 (90.9%)	
Lobectomy with Sleeve Resection	57 (0.1%)	21 (0.1%)	36 (0.1%)	
Segmentectomy	4955 (11.4%)	2720 (14.6%)	2235 (9.0%)	
**Case length [minutes] ± SD**	124 ± 70	136 ± 72	116 ± 67	< 0.001
**Da Vinci Model**				< 0.001
Xi	43,277 (99.5%)	18,604 (99.7%)	24,673 (99.3%)	
Non Xi	230 (0.5%)	60 (0.3%)	170 (0.7%)	
**Robotic Stapler Used**	36,901 (84.8%)	15,811 (84.7%)	21,090 (84.9%)	0.606
**Number of Stapler Applications**				0.002
0	6610 (15.2%)	2853 (15.3%)	3757 (15.1%)	
1–5	7359 (16.9%)	3026 (16.2%)	4333 (17.4%)	
6–10	17,986 (41.3%)	7710 (41.3%)	10,276 (41.4%)	
> 10	11,552 (26.6%)	5075 (27.2%)	6477 (26.1%)	
**Facility Type**				< 0.001
Academic/Teaching	14,300 (32.9%)	10,861 (58.2%)	3439 (13.8%)	
Community Hospital	28,221 (64.9%)	6942 (37.2%)	21,279 (85.7%)	
DOD/VA Hospital	986 (2.3%)	861 (4.6%)	125 (0.5%)	
**Census Region**				< 0.001
Midwest	9256 (21.3%)	4001 (21.4%)	5255 (21.2%)	
Northeast	10,957 (25.2%)	6361 (34.1%)	4596 (18.5%)	
South	16,505 (37.9%)	5653 (30.3%)	10,852 (43.7%)	
West	6789 (15.6%)	2649 (14.2%)	4140 (16.7%)

**Fig. 1:**
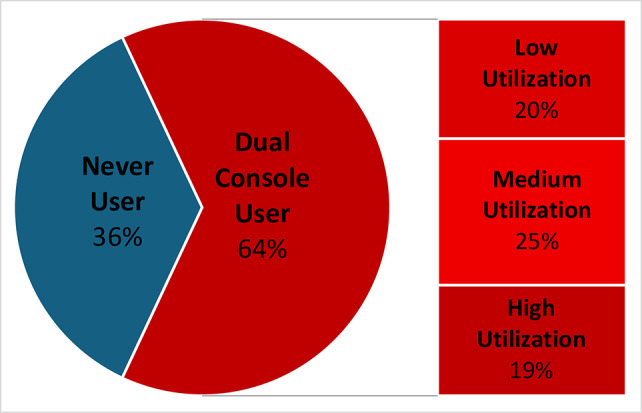
Pie chart illustrating the proportion of surgeons using the dual console for robotic-assisted anatomic lung resections

## Surgeon characteristics associated with dual console use

**Fig. 2:**
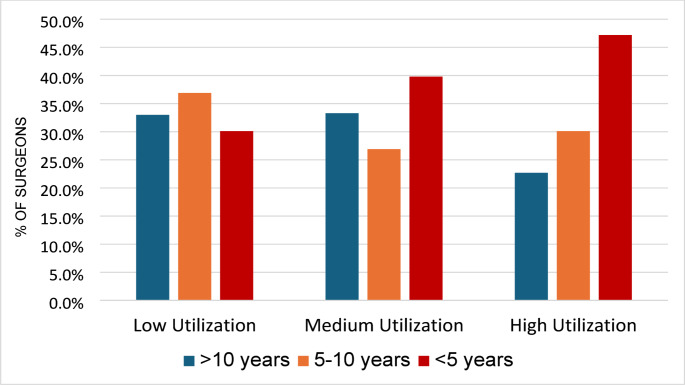
Frequency of dual console utilization in anatomic lung resection by years of experience of the primary surgeon

Of 868 surgeons who performed robotic-assisted anatomic lung resections, 555 (63.9%) utilized a DC at least once compared to 313 (36.1%) who never did (Fig. 1). The vast majority of surgeons performing anatomic lung resections were primarily thoracic surgeons (96.3%). Surgeons with DC cases were stratified into low (176, 31.7%), medium (216, 38.9%), and high (163, 29.3%) utilization. There was a significant association between surgeon experience level and frequency of DC utilization (Table 2). Medium or high utilization were more common amongst surgeons with < 5 years of DaVinci experience (49.8%) compared to surgeons with more than 10 years of experience (40.3%) (*p* = 0.018, Fig. 2). Operative volume measured in number of previously completed cases was also significantly associated with DC use (Table 1). The highest rate of never DC users performed less then 50 anatomic resection 44.1%, compared to 23.8% of that cohort having completed over 100 anatomic resections (Table 2). However, the proportion of high utilizers was similar between surgeons with less than 50 and more than 100 prior anatomic resections (18.1% vs. 15.8%, respectively). In addition, there was a significantly higher proportion of high DC utilizers in the Northeast (32.7%) compared to the Midwest (17.6), West (18.2%) or South (11.1%, *p* < 0.001, Table 2).

**Table 2:** Characteristics of surgeons performing anatomic lung resections based on dual console utilization

Surgeon Characteristics	*N*	Never DC User	Low DC Utilizer	Medium DC Utilizer	High DC Utilizer	*p*-Value
**Primary** **Specialty**						0.044
Thoracic	836	295 (35.3%)	169 (20.2%)	211(25.2%)	161 (19.3%)	
Cardiac	13	5 (38.4%)	4 (30.8%)	4 (30.8%)	0 (0.0%)	
General Surgery	19	13 (68.4%)	3 (15.8%)	1 (5.2%)	2 (10.5%)	
**Number of Prior DaVinci Anatomic Resections**						< 0.001
1–49	550	234 (44.1%)	96 (17.5%)	120 (21.9%)	100 (18.1%)	
50–99	217	55 (25.3%)	52 (24.0)	63 (29.0%)	47 (21.7%)	
100+	101	24 (23.8%)	28 (26.7%)	33 (32.7%)	16 (15.8%)	
**Advanced Robotic Training**						0.073
Prior to 2014	77	29 (37.6%)	20 (26.0%)	17 (22.1%)	11 (14.3%)	
2014–2019	437	177 (40.5%)	102 (23.3%)	95 (21.7%)	63 (14.4%)	
2020–2023	124	46 (37.1%)	25 (20.2%)	20 (16.1%)	33 (26.6%)	
**Length of Da Vinci Use experience**						0.018
< 5 Years	327	111 (33.9%)	53 (16.2%)	86 (26.3%)	77 (23.5%)	
5–10 Years	269	97 (36.1%)	65 (24.2%)	58 (21.6%)	49 (18.1%)	
> 10 Years	272	105 (38.6%)	58 (21.3%)	72 (26.7%)	37 (13.6%)	
**Census Region**						< 0.001
Midwest	188	64 (34.0%)	38 (20.2%)	53 (28.2%)	33 (17.6%)	
Northeast	202	45 (22.3%)	32 (15.8%)	59 (29.2%)	66 (32.7%)	
South	330	152 (46.1%)	68 (20.1%)	73 (22.1%)	37 (11.1%)	
West	148	52 (35.1%)	38 (25.7%)	31 (20.9%)	27 (18.2%)

### Independent predictors of dual console use

A multivariable model combining case and surgeon factors demonstrated factors that were independently associated with DC use (Table 3). Cases performed by surgeons with less than 5 years were significantly more likely to use the DC compared to surgeons with 5–10 years of robotic experience (odds ratio (OR) 1.62, 95% confidence interval (CI) 1.05–2.51, *p* = 0.031), but not different to surgeons with > 10 years of experience (Table 3). Additionally, cases at Academic/Teaching hospitals (odds ratio 6.02, 95% confidence interval 3.94–9.22, *p* < 0.001) or Department of Defense/Veterans Affairs hospitals hospital (OR 10.10, 95% CI 5.21–19.61, *p* < 0.001) were significantly more likely to have DC utilization compared to the cases performed at community hospitals. Additionally, a higher number of robotic stapler use was also associated with teaching console setup (Table 3).

**Table 3:** Multivariable analysis of dual console use

	Comparison	Odds Ratio (95% Confidence Interval)	*p*-Value
**Surgeon DaVinci Experience**	5–10 Years vs. <5 Years	0.617 (0.398–0.956)	0.031
	5–10 Years vs. > 10 Years	0.777 (0.495–1.220)	0.272
	< 5 Years vs. > 10 Years	1.259 (0.752–2.101)	0.382
**Number of Stapler Fires**	0 vs. 1–5	0.872 (0.736–1.034)	0.115
	0 vs. 6–10	0.836 (0.707–0.988)	0.035
	0 vs. > 10	0.758 (0.640–0.898)	0.001
	1–5 vs. 6–10	0.958 (0.900–1.020)	0.181
	1–5 vs. > 10	0.869 (0.805–0.938)	< 0.001
	6–10 vs. > 10	0.907 (0.855–0.962)	0.001
**Facility Type**	Academic/Teaching vs. Community Hospital	6.023 (3.936–9.216)	< 0.0001
	Academic/Teaching vs. DOD/VA Hospital	0.597 (0.351–1.016)	0.057
	Community Hospital vs. DOD/VA Hospital	0.099 (0.051–0.192)	< 0.0001

## Discussion

The teaching console is the key requirement for robotic surgery training in robotic surgery. This study is one of the first to examine national DC adoption for advanced thoracic robotic surgical procedures. In evaluating the frequency of DC use for anatomic lung resection on the daVinci system, we found that a significant proportion (42.9%) of robotic cases performed used this teaching setup. Consistent with a previous study, the use of the DC added approximately 18 min of console time on average [3]. Through the analysis of case and surgeon factors associated with the two surgeon DC use, we gained some insights into the teaching landscape in the US. We found that overall, approximately two-thirds of thoracic surgeons have used a DC setup during the study period, with variable frequency. The DC utilization was highest for cases performed by surgeons with less than 5 years of experience on the da Vinci, and surgery performed at hospitals classified as Academic/Teaching facilities.

As robotic thoracic surgery continues to grow, a DC provides the foundation to train console surgeons by facilitating real-time guidance, enabled by constant line of communication via microphone, and instructor camera overlaid virtual pointers to navigate trainee maneuvers, and instrument swapping between attending and trainee [4, 5]. A recent Society of Thoracic Surgery (STS) practice guideline highlighted the DC setup as an essential component for a standardized curriculum in robotic thoracic surgery [6]. Experts have recommended that increasing graduated autonomy on the DC appears to be the safest and most efficient way to train surgeons in robotic thoracic surgery [7]. The findings in the current study of surgeons with less than five years of robotic surgery experience employing higher utilization of the DC is possibly a representation of these surgeons’ own experiences in having been taught on the DC during their training and resulting in a willingness to teach via the DC. In a recent study, Wang and colleagues interviewed surgeons at a major academic medical center and found the two main reasons for reasons for faculty surgeons to grant resident autonomy on the DC was based on their assessment of resident training and their perceived pressure to complete the operation in a timely fashion [8]. Interestingly, the same study showed that neither the procedure type, nor the case volume for the teaching surgeon were associated with granting trainee autonomy on the robot [8]. 

There are mixed results in the literature regarding impact of DC use on case length and cost in colorectal, oncologic, and urological surgeries [9–11]. A recent study showed that teaching general surgery residents on the robot did not add additional time or operating room cost compared to teaching non-robotic cases when case complexity is low [12]. Kernstine and colleagues reported the largest study evaluating resident involvement during robotic surgery cases [13]. This study showed a gradual increase in the console involvement of trainees in 211 anatomic lung resections resulted in comparable short-term and long-term oncologic outcomes compared to resection done by VATS and open thoracotomy [13]. DC use thus allows the assistant to gradually develop experience with economy of motion in operating multiple instruments, obtain the same 3-dimension camera depth perception view as the primary surgeon, and gain familiarity with the lack of tactile feedback, all while preserving patient safety and efficiency in the operating room.

This study is important because it is the first to analyze what surgeon factors influence robotic DC use in thoracic surgery, which has important consequences for training the next generation of thoracic surgeons. The findings from this study accentuate the importance of DC use in surgical education, prominent in academic centers where trainee education is a core value. The spike in DC use in the Northeast region is likely a byproduct of a large concentration of academic centers in that region. Further, as DC use was associated with a higher number of stapler fires, it is possible that this could be a surrogate measure for increased case complexity. It is conceivable that surgeons who have the skills to perform more complex cases on the robot may also be more apt to teach trainees on the DC. On the other hand, case complexity may simply be higher in academic centers based in referral patterns. Robotic stapling also negates the need for assistant surgeons to navigate the manual stapler at the bedside and allows training surgeons to spend more time at the console.

The study has several strengths and limitations. The timeframe was based on data availability, which reflects contemporary practice of two recent years, but it does not include the early adoption of this technology. Low volume surgeons were also excluded from the analysis. DC use was analyzed based on any activation and does not account for the activation time and which parts of the cases were performed on the DC. The availability of a second console is not captured in the database, which may account for the variations in DC use and teaching practices between surgeons. There is also a lack of data on the rank of the DC user, their years in surgical training, and experience with robotic surgery. We assumed that the DC setup is used for surgeons in training, but actual use may include utilization by two surgeons in practice, in which the less experienced surgeon operates with a mentor, or cases in which another surgeon is asked to join for help or provide a second opinion. Conversely, surgeons may have taken turns with their trainees on a single console, which would not be captured as a teaching case. This study did not incorporate clinical data to understand the effects of case complexity or impact of DC use on postoperative patient outcomes.

## Conclusions

This study demonstrates widespread national adoption of DC for teaching robotic anatomic lung resection, with approximately 43% of cases leveraging this setup. The factors associated with increased DC use included surgeons with fewer years of robotic surgery experience, surgery performed at an academic hospital, and cases with greater number of robotic stapler applications. Further research is needed on robotic surgery education by understanding the impact of DC use on patient outcomes and trainee learning curves to pave the path for the next generation of minimally invasive thoracic surgeons.

## Data Availability

Data is provided within the manuscript.

## References

[1] D’Souza DM, Kneuertz PJ, Cheufou D (2024) The 7 pillars of proctorship in robotic thoracic surgery: A blueprint for a successful start. Innovations (Phila) 19(5):459–463. 10.1177/15569845241285361 39382128 10.1177/15569845241285361

[2] Schmiederer S, Torices-Dardon I, Ferrari-Light AM (2021) Developing a robotic general surgery training curriculum: identifying key elements through a Delphi process. J Surg Educ 78(6):e129–e136. 10.1016/j.jsurg.2021.08.006 34456170 10.1016/j.jsurg.2021.08.006

[3] Vijayakumar A, Abdel-Rasoul M, Hekmat R et al (2024) National learning curves among robotic thoracic surgeons in the United States: quantifying the impact of procedural experience on efficiency and productivity gains. J Thorac Cardiovasc Surg 167(3):869-879e2. 10.1016/j.jtcvs.2023.07.051 37562675 10.1016/j.jtcvs.2023.07.051

[4] Fernandes E, Elli E, Giulianotti P (2014) The role of the dual console in robotic surgical training. Surgery 155(1):1–4. 10.1016/j.surg.2013.06.023 23973110 10.1016/j.surg.2013.06.023

[5] Zirafa CC, Romano G, Key TH, Davini F, Melfi F (2019) The evolution of robotic thoracic surgery. Ann Cardiothorac Surg 8(2):210–217. 10.21037/acs.2019.03.03 31032204 10.21037/acs.2019.03.03 PMC6462549

[6] Kim SS, Schumacher L, Cooke DT et al (2025) The society of thoracic surgeons expert consensus statements on a framework for a standardized national robotic curriculum for thoracic surgery trainees. Ann Thorac Surg 119(4):719–732. 10.1016/j.athoracsur.2024.12.003 39706508 10.1016/j.athoracsur.2024.12.003

[7] Mitzman B, Smith BK, Varghese TK (2023) Resident training in robotic thoracic surgery. Thorac Surg Clin 33(1):25–32. 10.1016/j.thorsurg.2022.07.009 36372530 10.1016/j.thorsurg.2022.07.009

[8] Wang TN, Woelfel IA, Huang E, Pieper H, Meara MP, Chen XP (2024) Behind the pattern: general surgery residsent autonomy in robotic surgery. Heliyon Jun 15(11):e31691. 10.1016/j.heliyon.2024.e31691 10.1016/j.heliyon.2024.e31691 PMC11152925 38841510

[9] Bolger JC, Broe MP, Zarog MA et al (2017) Initial experience with a dual-console robotic-assisted platform for training in colorectal surgery. Tech Coloproctol 21(9):721–727. 10.1007/s10151-017-1687-8 28929257 10.1007/s10151-017-1687-8

[10] De Pastena M, Salvia R, Paiella S et al (2021) Robotic dual-console distal pancreatectomy: could it be considered a safe approach and surgical teaching even in pancreatic surgery? A retrospective observational study cohort. World J Surg 45(10):3191–3197. 10.1007/s00268-021-06216-y 34304274 10.1007/s00268-021-06216-y PMC8408081

[11] Morgan MS, Shakir NA, Garcia-Gil M et al (2015) Single- versus dual-console robot-assisted radical prostatectomy: impact on intraoperative and postoperative outcomes in a teaching institution. World J Urol 33(6):781–786. 10.1007/s00345-014-1349-7 24973046 10.1007/s00345-014-1349-7

[12] Chen X, Meara M, Harzman A, Pieper H, Ellison EC (2023) Cost analysis of training residents in robotic-assisted surgery. Surg Endosc 37(4):2765–2769. 10.1007/s00464-022-09794-7 36471060 10.1007/s00464-022-09794-7

[13] Nawalaniec JT, Elson M, Reznik SI et al (2022) Training cardiothoracic residents in robotic lobectomy is cost-effective with no change in clinical outcomes. Innovations (Phila) 17(2):127–135. 10.1177/15569845221086278 35341368 10.1177/15569845221086278

